# Visceral and subcutaneous adipose tissue in children born after ART with frozen and fresh embryo transfers

**DOI:** 10.1093/hropen/hoaf014

**Published:** 2025-03-17

**Authors:** Annesofie R Olsen, Louise L Asserhøj, Anja Pinborg, Tine D Clausen, Gorm Greisen, Rikke B Jensen, Katharina M Main, Niels G Vejlstrup, Per L Madsen, Ikram Mizrak

**Affiliations:** Department of Cardiology, Copenhagen University Hospital—Herlev and Gentofte Hospital, Herlev, Denmark; The Fertility Clinic, Department of Gynaecology, Fertility and Obstetrics, Centre JMC, Copenhagen University Hospital—Rigshospitalet, Copenhagen, Denmark; The Fertility Clinic, Department of Gynaecology, Fertility and Obstetrics, Centre JMC, Copenhagen University Hospital—Rigshospitalet, Copenhagen, Denmark; The Fertility Clinic, Department of Gynaecology, Fertility and Obstetrics, Centre JMC, Copenhagen University Hospital—Rigshospitalet, Copenhagen, Denmark; Department of Clinical Medicine, University of Copenhagen, Copenhagen, Denmark; Department of Clinical Medicine, University of Copenhagen, Copenhagen, Denmark; Department of Gynaecology and Obstetrics, The North Zealand Hospital, Hilleroed, Denmark; Department of Clinical Medicine, University of Copenhagen, Copenhagen, Denmark; Department of Neonatology, Copenhagen University Hospital—Rigshospitalet, Copenhagen, Denmark; Department of Clinical Medicine, University of Copenhagen, Copenhagen, Denmark; Department of Paediatrics, Copenhagen University Hospital—Herlev and Gentofte Hospital, Herlev, Denmark; Department of Clinical Medicine, University of Copenhagen, Copenhagen, Denmark; Department of Growth and Reproduction, Copenhagen University Hospital—Rigshospitalet, Copenhagen, Denmark; International Centre for Research & Training in Disruption of Male Reproduction & Child Health (EDMaRC), Copenhagen University Hospital—Rigshospitalet, Copenhagen, Denmark; Department of Cardiology, Copenhagen University Hospital—Rigshospitalet, Copenhagen, Denmark; Department of Cardiology, Copenhagen University Hospital—Herlev and Gentofte Hospital, Herlev, Denmark; Department of Clinical Medicine, University of Copenhagen, Copenhagen, Denmark; The August Krogh Institute, NEXS, University of Copenhagen, Copenhagen, Denmark; Department of Cardiology, Copenhagen University Hospital—Herlev and Gentofte Hospital, Herlev, Denmark; The Fertility Clinic, Department of Gynaecology, Fertility and Obstetrics, Centre JMC, Copenhagen University Hospital—Rigshospitalet, Copenhagen, Denmark

**Keywords:** cardiometabolic health, MRI, visceral and subcutaneous adipose tissue, frozen embryo transfer, fresh embryo transfer

## Abstract

**STUDY QUESTION:**

Is the ratio of visceral adipose tissue (VAT) and subcutaneous adipose tissue (SAT) comparable between children following ART and natural conception (NC)?

**SUMMARY ANSWER:**

Children conceived by frozen embryo transfer (FET) had slightly lower VAT/SAT ratios than children following NC; no difference in VAT/SAT ratio was observed in children born following fresh embryo transfer (Fresh-ET) as compared to those born from NC.

**WHAT IS KNOWN ALREADY:**

The VAT/SAT ratio is closely related to the metabolic profile, with a high ratio increasing the risk of cardiometabolic diseases. To our knowledge, no studies have reported the VAT/SAT ratio in children following ART.

**STUDY DESIGN, SIZE, DURATION:**

This prospective exploratory observational cohort study included 150 singletons aged 7–10 years. All children were born in eastern Denmark. The study was conducted between November 2018 and August 2020.

**PARTICIPANTS/MATERIALS, SETTING, METHODS:**

This is a sub-study of the ‘Health in Childhood following Assisted Reproductive Technology’ (HiCART) study. The children were conceived after FET (n = 50), Fresh-ET (n = 50), and NC (n = 50), and children conceived by NC were matched to ART children by sex and birth year. The children underwent abdominal MRI for the quantification of abdominal adipose tissues along with measurements of blood pressure, fasting blood samples, anthropometric measurements, and dual-energy X-ray absorptiometry scans. The volumes of VAT and SAT were semi-automatically quantified, blinded for the mode of conception. The level of statistical significance was set to a *P*-level below 0.05. Multivariable linear regression analysis of the VAT/SAT ratio was performed to adjust for confounders in a five-step approach: Model 1: Adjusted for child age and sex; Model 2: Model 1 plus maternal age at delivery and maternal BMI at pregnancy; Model 3: Model 2 plus birth weight and child BMI; Model 4a: Model 3 plus maternal educational level; Model 4b: Model 3 plus pubertal status. The confounders were selected based on their association with metabolic risk factors according to previous studies.

**MAIN RESULTS AND THE ROLE OF CHANCE:**

As previously reported in the HiCART studies, there were no differences between the groups in anthropometric measurements including BMI, lean body mass, blood pressure, or triglycerides. The crude VAT/SAT ratio differed significantly between the three groups (mean (SD); FET 0.26 (0.08), Fresh-ET 0.29 (0.07), NC 0.30 (0.08), ANOVA—*P* = 0.014). Pairwise comparison revealed that children conceived after FET had lower crude VAT/SAT ratio than children conceived after NC (*P* = 0.007) with a mean difference of −0.04, 95% CI (−0.07; −0.01), and a tendency for a lower VAT/SAT ratio as compared to the Fresh-ET group (*P* = 0.059) with a mean difference of −0.03, 95% CI (−0.06; 0.00). Lower VAT/SAT ratio in FET as compared to NC remained after adjustment for child age and sex (Model 1: −0.04 (−0.07; −0.01)), maternal age at delivery and maternal BMI at pregnancy (Model 2: −0.04 (−0.07; −0.01)), birth weight and child BMI (Model 3: −0.04 (−0.07; −0.01)), maternal educational level (Model 4a: −0.05 (−0.08; −0.01)), and puberty (Model 4b: −0.04 (−0.08; −0.01)) in a five-step approach. Repeated analysis of twenty MRI scans showed good intra-rater repeatability of VAT and SAT volume quantifications.

**LIMITATIONS, REASONS FOR CAUTION:**

The sample size was relatively small and selection bias due to differences in intrinsic factors between the three groups may affect the results. Well-described confounders from the literature were included in the multivariable regression analysis, but the observational nature of this cohort study hinders the establishment of causality.

**WIDER IMPLICATIONS OF THE FINDINGS:**

Reassuringly, this study found no clinically important difference in VAT/SAT ratio between children following ART (both FET and Fresh-ET) and NC, although a small but significantly lower VAT/SAT ratio was found in children born after FET compared with NC children.

**STUDY FUNDING/COMPETING INTEREST(S):**

A.R.O was supported by a scholarship from Herlev-Gentofte Copenhagen University Hospital. The study was funded by grants from Novo Nordisk Foundation (NNF18OC0034092, NFF19OC0054340) and The Research Foundations at Rigshospitalet and Herlev-Gentofte Copenhagen University Hospital (unrestricted grant). A.P. has received grants (via her institution), honoraria, and consulting fees from Gedeon Richter, Ferring Pharmaceuticals, and Merck A/S, as well as consulting fees from Novo Nordisk A/S and Cryos, honoraria from Organon and support for attending meetings (via her institution) from Gedeon Richter. K.M.M. has received royalties from Gyldendal and consulting fees from The National Board of Wealth and Welfare in Sweden, in addition to honoraria from Novo Nordisk A/S and Lundbeck A/S, and serves as a medical expert for the Ministry of Justice, Department of Civil Affairs. All other authors declare no conflicts of interest.

**TRIAL REGISTRATION NUMBER:**

ClinicalTrials.gov identifier: NCT03719703.

WHAT DOES THIS MEAN FOR PATIENTS? This study looks at whether children conceived by assisted reproductive technology, both frozen and fresh embryo transfer, have abnormally distributed abdominal fat as compared to naturally conceived children. More specifically, we looked at the ratio between visceral fat (located around the internal organs) and subcutaneous fat (located under the skin) in the whole abdomen using the reference standard of magnetic resonance imaging (MRI).

Research indicates that a higher amount of visceral fat as compared to subcutaneous fat is associated with metabolic problems related to obesity. According to previous research, children conceived by frozen and fresh embryo transfer may be at risk of obesity due to their risk of being born large- and small-for-gestational-age, respectively.

As the use of assisted reproduction continues to rise in numbers, it is important to evaluate the safety of the procedure in relation to potential health consequences. In this study, children conceived by assisted reproductive technology using frozen embryo transfer were found to have a lower visceral-to-subcutaneous fat ratio as compared to children conceived naturally; however, this is not believed to be of clinical relevance. There was no difference between children born after fresh embryo transfer and those conceived naturally. These findings are reassuring for the future use of assisted reproductive technology.

## Introduction

Studies suggest that cardiometabolic risk factors may be increased in children born after ARTs and adverse fat distribution may be a causal factor (Ceelen *et al.*, 2007, 2008; Berntsen *et al.*, 2019; Valenzuela-Alcaraz *et al.*, 2019; Norrman *et al.*, 2020, 2021; Boutet *et al.*, 2021). The distribution of the abdominal adipose tissue is a biological marker of the metabolic profile that is closely associated with the risk of cardiometabolic diseases (Kelly *et al.*, 2014; Yan *et al.*, 2019). Notably, increased visceral adipose tissue (VAT) is associated with insulin resistance and subsequently increased risk of hypertension and type 2 diabetes mellitus (Fox *et al.*, 2007; Abraham *et al.*, 2015) whereas abdominal subcutaneous adipose tissue (SAT) has been found to be inversely associated with insulin resistance and thus have a neutral or potentially advantageous association with glucose metabolism (McLaughlin *et al.*, 2011). VAT is more strongly correlated with cardiometabolic risk factors than SAT and total adipose tissue (TAT), as increased VAT volume is associated with chronic low-grade inflammation through the secretion of pro-inflammatory cytokines (Fox *et al.*, 2007; Bays *et al.*, 2008; Ibrahim, 2010; Ouchi *et al.*, 2011; Britton *et al.*, 2013; Rospleszcz *et al.*, 2019; Hildebrandt *et al.*, 2023). However, the VAT/SAT ratio as compared to the total VAT volume, is believed to be an important marker for cardiometabolic health as it is reported to be a better independent predictor of mortality and cardiac events (Ladeiras-Lopes *et al.*, 2017).

ART treatments continue to rise in numbers, and ART offspring now constitutes up to 8% of the annual birth cohorts in Europe (European IVF Monitoring Consortium (EIM) for the European Society of Human Reproduction and Embryology (ESHRE) *et al.*, 2022, 2023; Pinborg *et al.*, 2023). Hence, the health of ART offspring is pivotal. Children born after ART with frozen embryo transfer (FET), or fresh embryo transfer (Fresh-ET) are prone to be born large- and small-for-gestational-age, respectively (Maheshwari *et al.*, 2012; Pinborg *et al.*, 2014; Asserhøj *et al.*, 2021; Luke *et al.*, 2021; Terho *et al.*, 2021a). Previous studies have reported that being born both large- and small-for-gestational-age is associated with obesity and adverse abdominal fat distribution (Kaul *et al.*, 2019; Zhang *et al.*, 2024). Moreover, obesity and adverse abdominal fat distribution may potentially affect cardiometabolic health later in life (Nordman *et al.*, 2020; Magnusson *et al.*, 2021; Bizerea-Moga *et al.*, 2022). In this study, we used the non-invasive reference standard of MRI to investigate the hypothesis that children conceived after FET and Fresh-ET have higher VAT/SAT ratios as compared to children conceived after natural conception (NC). Also, we aimed to explore whether the VAT/SAT ratio was correlated with triglycerides and high-sensitive C-reactive protein (hs-CRP).

## Materials and methods

### Ethics approval

The Scientific Ethics Committee of the Capital Region (H-18000122) and the Danish Protection Agency (VD-2018-310) approved the study protocol. This study was conducted in accordance with The Helsinki Declaration and informed written consent was collected from all custody-holding parents.

### Study participants and design

This is an exploratory observational cohort study of 150 singletons aged 7–10 years, as the study is a sub-study of the HiCART (*Health in Childhood following Assisted Reproductive Technology*) cohort study initiated in 2018. The children were conceived after FET (n = 50), Fresh-ET (n = 50), or NC (n = 50). The children were identified through their mother’s civil registration number in the Danish In-Vitro Fertilization Registry and the Medical Birth Registry. The recruitment procedure and sample size calculation, determined through a power analysis based on an anticipated difference in aortic distensibility, have been previously described (Mizrak *et al.*, 2022b). Only singletons and children born in the Capital and Zealand regions of Denmark were included. We excluded children of mothers with gestational diabetes or diabetes mellitus, children conceived through oocyte donation, as well as children with known congenital heart disease or contraindications for an MRI scan. Children conceived naturally were matched to children born after ART by sex and birth year. All children underwent an abdominal MRI along with measurements of blood pressure, fasting blood samples including hs-CRP, anthropometric measurements, and a dual-energy X-ray absorptiometry (DXA) scan to determine fat-free mass. Moreover, neonatal data and parental characteristics were collected. Educational level was collected from the maternal questionnaire. Long-term higher educational level was defined as more than 16 years of education. The study design and baseline characteristics of the participants have previously been described in detail (Mizrak *et al.*, 2022a,b). Also, biochemical markers of glucose- and lipid metabolism of the studied population and neonatal data have previously been published (Mizrak *et al.*, 2022a,b). All examinations and analyses were performed blinded to the mode of conception.

### Non-participants

A non-participant analysis was conducted to examine differences between participants and non-participants. A more elaborate description of the non-participant analysis has previously been published (Mizrak *et al.*, 2022b).

### Abdominal adipose tissue MRI

All children underwent abdominal MRI scan in a 1.5-Tesla MR scanner (MAGNETOM Aera; Siemens Healthcare, Erlangen, Germany) for quantification of abdominal adipose tissues. The children and their parents participated in an ∼1 h introduction to the MRI scan, and one parent sat close to the child during the MRI scan. No anaesthetics or contrast agents were administered. An 18- and a 4-channel phased-array coils were used to fully cover the area of interest. All cross-sectional abdominal MRI images were acquired with a field of view of 320 mm × 260 mm, voxel size 1.3 mm × 1.3 mm × 3.00 mm, TE_1_ (time to echo)/TE_2_ = 2.39 ms/4.77 ms, TR (time of repetition) = 6.68 ms. Images included the entire abdomen and were obtained between the level of Th_12_/L_1_ to L_5_/the sacral promontory using two-point Dixon fat–water separation during breath-holds. Dixon fat–water separation is acquired from in- and opposed-phase sequences, and from these, fat- and water-only images are computed (Simoni *et al.*, 2020). Hence, each abdominal scan consisted of ∼60 slices. We aimed to cover the complete volume of abdominal adipose tissue but also expressed single-slice adipose tissue at the vertebral level L_3_ to make our data relatable to other single-slice studies and to test intra-rater repeatability.

### Quantification of abdominal adipose tissue

**Figure 1.**
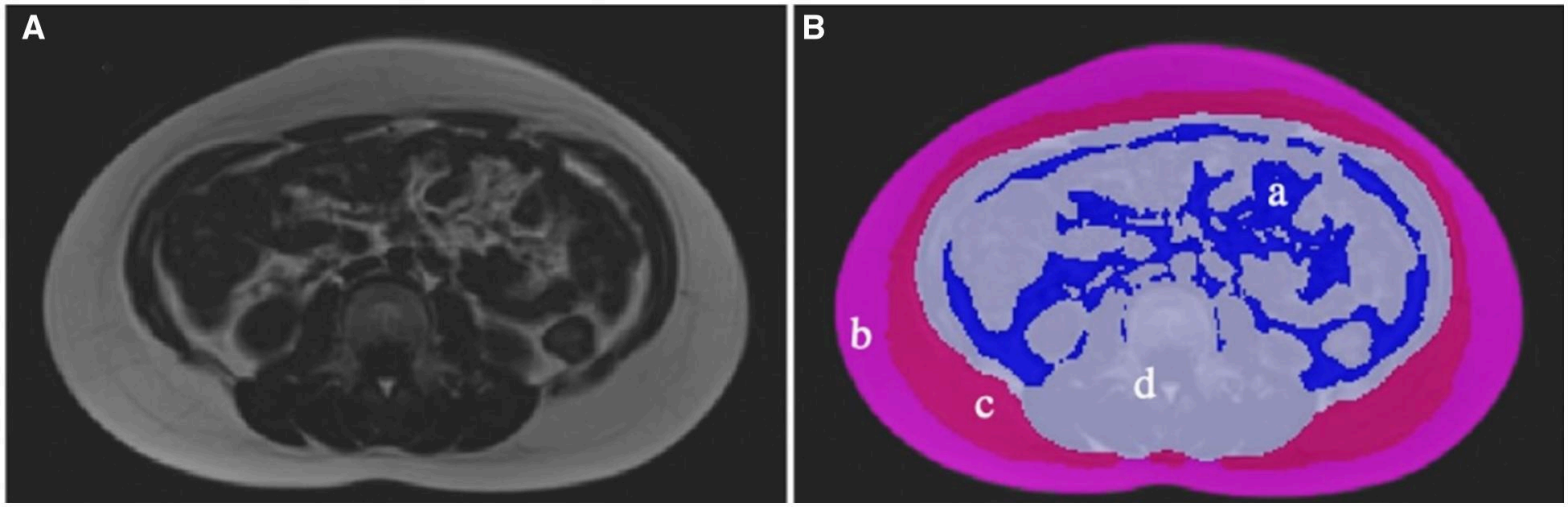
**Cross-sectional abdominal MRI for adipose tissue quantification (level L_3_).** The participant was a 9-year-old girl with BMI of 25.2. (**A**) Fat-only Dixon MRI at the level of L_3_. (**B**) Quantifiable adipose tissue based on Image **A** at the level of L_3_; a (blue) visceral adipose tissue; b (light pink) superficial subcutaneous adipose tissue; c (dark pink) deep subcutaneous adipose tissue; d (grey) non-adipose tissue. L_3_, lumbar spine level L_3_.

The volumes of VAT, SAT, and non-adipose tissue (NAT) were semi-automatically quantified from the scans with commercially available software (SliceOmatic, version 5.0 Rev-10b, Tomovision Inc., Montreal, Canada) based on signal intensity of adipose tissue. After the semi-automatic differentiation of the abdominal tissue, all data were visually revised and corrected by one trained observer blinded with respect to study groups. The subcutaneous fat located externally from the abdominal muscles within the abdominal region was defined as SAT. According to the position of the fascia superficialis, the SAT was further divided into superficial-SAT (SSAT) and deep-SAT (DSAT). The fat tissue located within the abdominal cavity was defined as VAT. The NAT was defined as the non-segmented area of residual organs (Fig. 1). SAT was determined as the sum of SSAT and DSAT. TAT was determined as the sum of SAT and VAT. The VAT/SAT ratio was calculated by dividing the volume of VAT by the volume of SAT.

### Statistical analyses

Statistical analyses were performed using R (version 4.4.1, R Core Team, 2020). All statistical analyses were pre-specified in an analysis plan. Statistical comparisons were performed from both parametric and non-parametric tests based on the appropriateness of the distribution of data. The comparison between study groups was assessed using one-way ANOVA, Kruskal–Wallis, or the χ^2^ test as appropriate. For pairwise comparisons, either *t*-test or Wilcoxon two-sample test was applied. The effect size for the groups was compared by calculating mean differences with 95% CI for continuous variables using univariable linear regression model. Data were expressed as mean (SD), median [interquartile ranges, IQR], or frequencies (percent, %) as appropriate. The level of statistical significance was set to a *P*-level below 0.05. Multivariable linear regression analysis was conducted to account for potential confounders in a five-step approach: Model 1: Adjusted for child age and sex; Model 2: Model 1 plus maternal age at delivery and maternal BMI at pregnancy; Model 3: Model 2 plus birth weight and child BMI; Model 4a: Model 3 plus maternal educational level; Model 4b: Model 3 plus pubertal status. The criteria for selection of these confounders were based on previous studies in which an association between ART treatments and metabolic syndrome were reported. Univariable linear regression analysis was conducted to analyse the association between VAT/SAT ratio and both log-transformed triglycerides and hs-CRP. The output of linear regression analysis is the regression coefficient and its respective 95% CI. The regression coefficient represents the estimated difference (beta-estimate) in the dependent variable for a one-unit change in the independent variable. Intra-rater analysis repeatability was tested in twenty randomly selected participants using Bland and Altman’s method. The repeated analysis was performed more than two months later, on single-slice images at the level of $L_{3}.$

## Results

### Participants and maternal characteristics

In this study, only selected and relevant background characteristics were included from the HiCART cohort. Child age was comparable between the three study groups (FET 8.8 years [8.6, 9.1], Fresh-ET 9.0 years [8.5, 9.2], NC 9.0 years [8.7, 9.2], Kruskal–Wallis—*P* = 0.643, Table 1) and 25 girls were included in each group. Child BMI, waist–hip ratio, and total fat-free mass did not differ significantly between the three groups (Table 1). According to our previously published data based on the HiCART cohort, neonatal data demonstrated an association between mode of conception and birth weight (FET 3772 g (485), Fresh-ET 3438 g (495), NC 3483 g (482), ANOVA—*P* = 0.001 and FET 0.4 SDS (1.1), Fresh-ET −0.1 SDS (0.1), NC −0.2 SDS (1.1), ANOVA—*P* = 0.020, Table 1). The number of children delivered before 37 weeks showed no significant difference between the three groups. Mothers receiving ART treatment were significantly older compared to mothers of children conceived naturally (FET 37 years (7), Fresh-ET 34 years (6), NC 33 years (5), ANOVA—*P* = 0.005, Table 1). Maternal BMIs at early pregnancy were comparable between the three groups (Table 1).

**Table 1.** Clinical and maternal characteristics of the three groups.

Variable	FET (n = 50)	Fresh-ET (n = 50)	NC (n = 50)	*P*-value
**Clinical characteristics**				
Age, median [IQR], years	8.8 [8.6, 9.1]	9.0 [8.5, 9.2]	9.0 [8.7, 9.2]	0.643^a^
Sex, girls	25 (50)	25 (50)	25 (50)	
Height, mean (SD), cm	138 (7)	137 (6)	137 (6)	0.708^b^
Weight, mean (SD), kg	31.9 (5.3)	31.6 (5.8)	31.0 (5.0)	0.724^b^
BMI at examination, mean (SD), kg/m^2^	16.7 (2.2)	16.8 (2.3)	16.5 (2.0)	0.825^b^
Waist–hip ratio, mean (SD)	0.85 (0.07)	0.82 (0.04)	0.84 (0.05)	0.137^b^
Total fat-free mass, mean (SD), kg	23.5 (3.6)	22.6 (3.1)	22.7 (3.0)	0.330^b^
Entered puberty, n (%)	6 (12)	7 (14)	7 (14)	0.950^c^
**Neonatal characteristics**				
Birth weight, mean (SD), g	3772 (485)	3438 (495)	3483 (482)	**0.001** ^b^
Birth weight, mean (SD), SDS	0.4 (1.1)	−0.1 (0.1)	−0.2 (1.1)	**0.020** ^b^
**Maternal characteristics**				
Number of mothers, n (%)	48 (96)	49 (98)	49 (98)	
Maternal age at delivery, mean (SD), years	37 (7)	34 (6)	33 (5)	**0.005** ^b^
Maternal BMI at early pregnancy, kg/m^2^	22.2 [21.0, 23.9]	23.3 [21.5, 26.6]	22.3 [21.1, 25.2]	0.381^a^
Educational level, n (%)				
Long-term higher education	23 (50)	27 (56)	15 (31)	**0.030** ^c^

Values are showed as mean (SD) or median [IQR, interquartile ranges]. Boldface indicates significant values. FET, frozen embryo transfer; Fresh-ET, fresh embryo transfer; NC, natural conception.

a Data were analysed using Kruskal–Wallis.

b Data were analysed using ANOVA.

c Data were analysed using χ^2^ test.

### Assessment of abdominal adipose tissue

**Figure 2.**
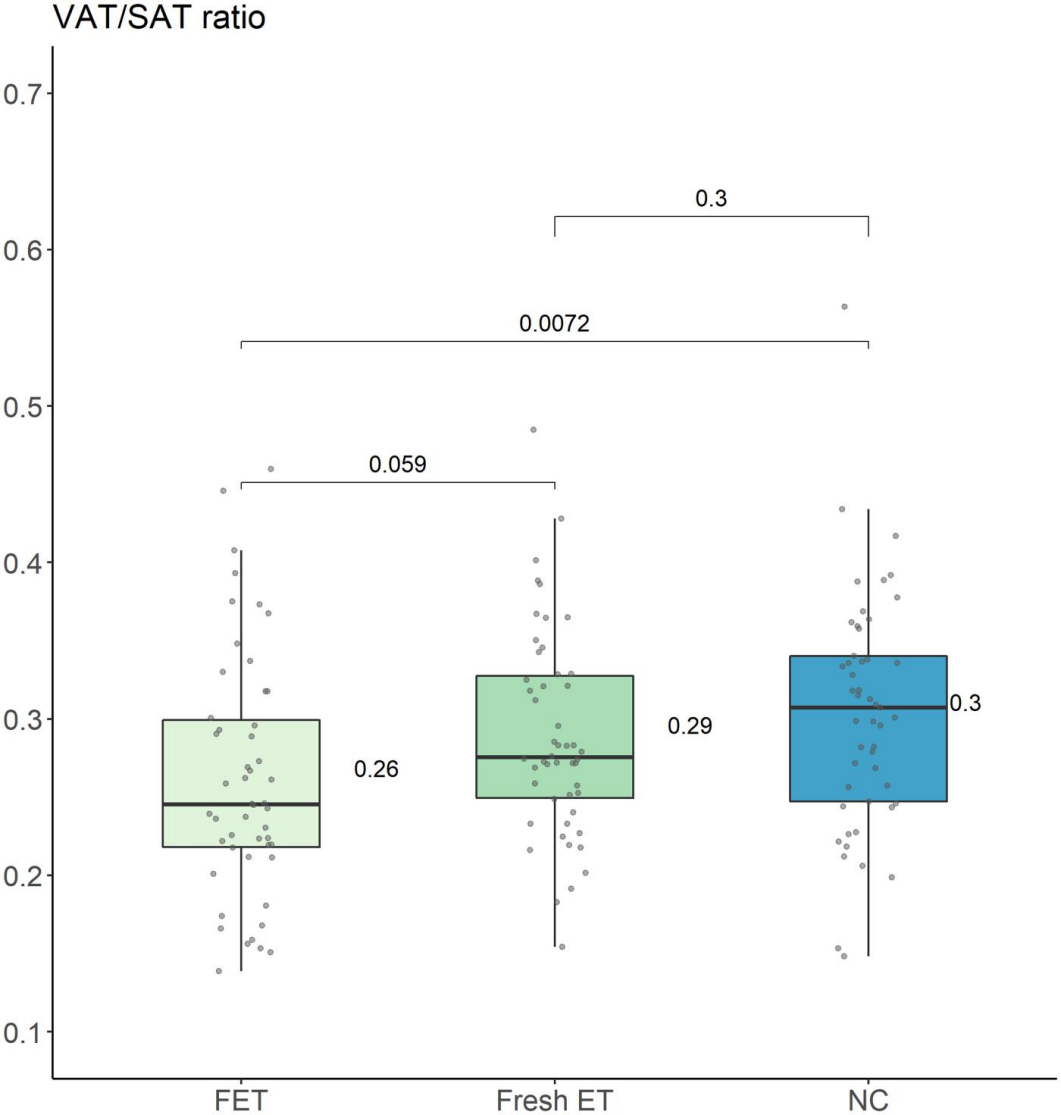
**Pairwise comparison of the VAT/SAT ratio using *t*-test.** Box-plot illustrating the mean VAT/SAT ratio of each group and pairwise comparisons of the VAT/SAT ratios among the three groups. VAT, visceral adipose tissue; SAT, subcutaneous adipose tissue; FET, frozen embryo transfer; Fresh-ET, fresh embryo transfer; NC, natural conception.

SAT and VAT values were slightly higher in Fresh-ET as compared to FET and NC children, but without reaching statistical significance (Table 2). The VAT/SAT ratio, however, was statistically different between the three groups (mean (SD); FET 0.26 (0.08), Fresh-ET 0.29 (0.07), NC 0.30 (0.08), ANOVA—*P* = 0.014, Table 2) driven by a lower VAT/SAT ratio in FET than in NC children (mean difference (95% CI); −0.04 (−0.07; −0.01), *t*-test—*P* = 0.007) with a tendency to be lower in FET than in Fresh-ET children (−0.03 (−0.06; 0.00), *t*-test—*P* = 0.059, Table 2 and Fig. 2).

**Table 2.** Quantification of abdominal adipose tissue.

Variable	FET (n = 50)	Fresh ET (n = 50)	NC (n = 50)	*P*-value	FET vs NC (crude)*	Fresh-ET vs NC (crude)*	FET vs Fresh-ET (crude)*
SAT, median [IQR], cm^2^	2130 [1712, 2941]	2200 [1863, 3699]	2060 [1745, 2518]	0.671^a^	23.76 (−622.84; 670.36)	299.22 (−347.38; 945.82)	−275.50 (−918.79; 367.86)
Superficial SAT, median [IQR], cm^2^	1812 [1547, 2234]	1828 [1597, 2666]	1719 [1541, 2069]	0.638^a^	3.79 (−301.68; 309.25)	136.37 (−169.10; 441.84)	−132.60 (−436.50; 171.34)
Deep SAT, median [IQR], cm^2^	325 [166, 737]	401 [234, 1000]	340 [215, 562]	0.600^a^	19.97 (−331.44; 371.38)	162.85 (−188.56; 514.27)	−142.90 (−492.52; 206.75)
VAT, median [IQR], cm^2^	556 [371, 926]	711 [487, 1099]	644 [484, 846]	0.179^a^	−84.93 (−285.15; 115.29)	1.36 (−198.86; 201.58)	−86.29 (−285.50; 112.92)
VAT/SAT ratio, mean (SD)	0.26 (0.08)	0.29 (0.07)	0.30 (0.08)	**0.014** ^b^	**−0.04 (−0.07; −0.01)**	−0.01 (−0.04; 0.01)	−0.03 (−0.06; 0.00)
TAT, median [IQR], cm^2^	2772 [2128, 3587]	2867 [2396, 4700]	2701 [2284, 3364]	0.594^a^	−61.17 (−891.02; 768.67)	300.58 (−529.27; 1130.44)	−361.80 (−1187.40; 463.90)
NAT, median [IQR], cm^2^	11 043 [10 332, 11 925]	10 949 [9646, 11 766]	10 546 [10 022, 11 660]	0.321^a^	475.30 (−158.76; 1109.30)	256.6 (−377.40; 890.66)	218.6 (−412.18; 849.46)

Values are showed as mean (SD) for normally distributed or median [IQR, interquartile ranges] for non-normally distributed data. Boldface indicates significant values. VAT, visceral adipose tissue; SAT, subcutaneous adipose tissue; TAT, total adipose tissue; NAT, non-adipose tissue; FET, frozen embryo transfer; Fresh-ET, fresh embryo transfer; NC, natural conception.

a Data were analysed using Kruskal–Wallis.

b Data were analysed using ANOVA.

* Pairwise comparison of groups by calculating mean differences with 95% CI for continuous variables (using univariable linear regression model).

The subsequent analysis limited to the L_3_ level, also showed a significant difference in VAT/SAT ratio between the three groups (Supplementary Table S1).

**Figure 3.**
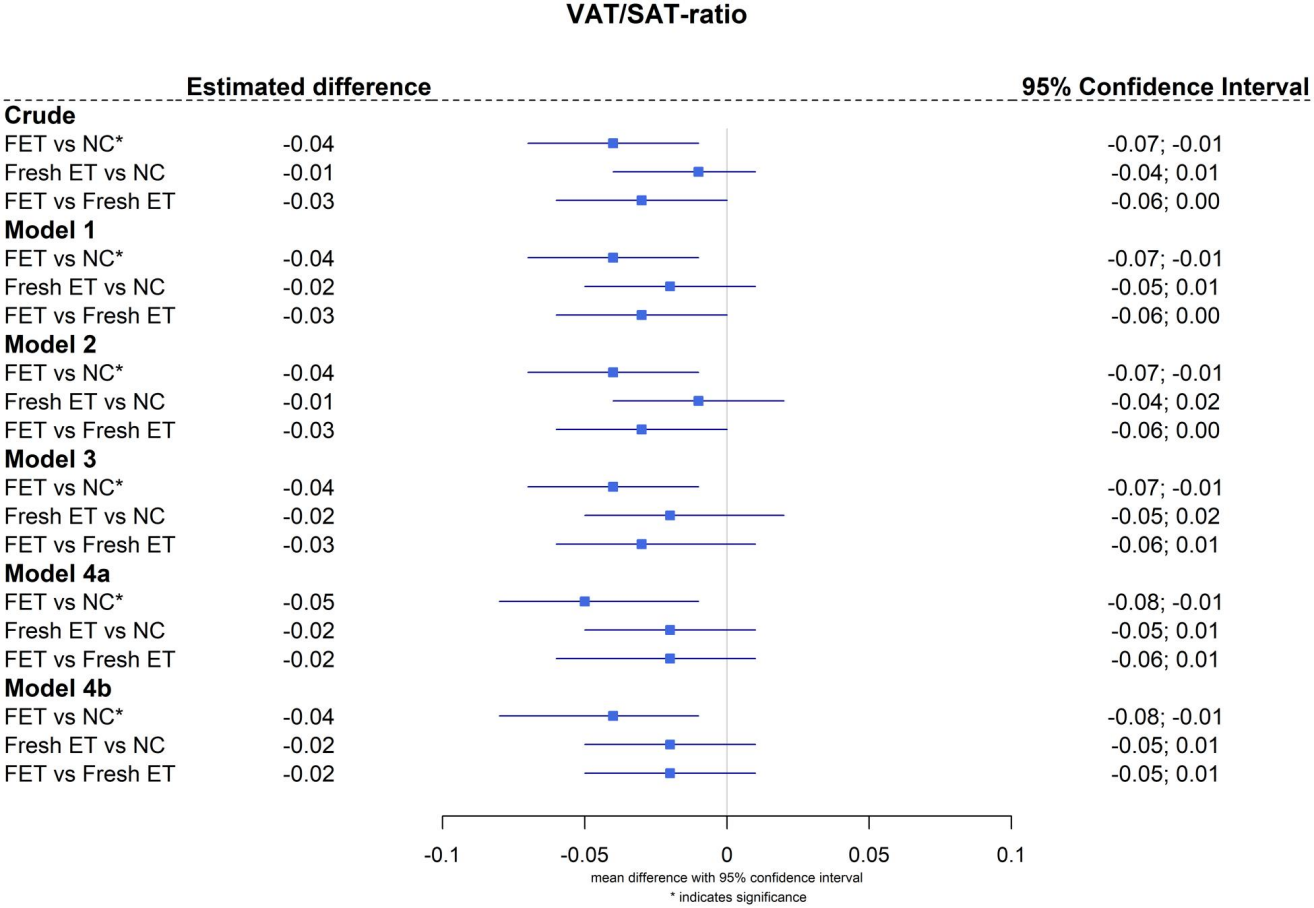
**Forest plot for multiple linear regression models for VAT/SAT ratio.** Presented data are estimated difference (beta-estimate) and 95% CI from multivariable linear regression analysis presented as a forest plot. Asterisk indicates significant values. Model 1: Adjusted for child age and sex. Model 2: Model 1 plus maternal age at delivery and maternal BMI at pregnancy. Model 3: Model 2 plus birth weight and child BMI. Model 4a: Model 3 plus maternal educational level. Model 4b: Model 3 plus pubertal status. VAT, visceral adipose tissue; SAT, subcutaneous adipose tissue; FET, frozen embryo transfer; Fresh-ET, fresh embryo transfer; NC, natural conception.

The lower VAT/SAT ratio in FET as compared to NC remained after multivariable regression analysis. Adjustments were made for child age and sex (−0.04 (−0.07; −0.01)), maternal age at delivery and maternal BMI at pregnancy (−0.04 (−0.07; −0.01)), birth weight and child BMI (−0.04 (−0.07; −0.01)), maternal educational level (−0.05 (−0.08; −0.01)), and puberty (−0.04 (−0.08; −0.01)) in a five-step approach (Fig. 3). Based on previously published data of triglycerides, we performed an additional correlation analysis between VAT/SAT ratio and logarithmically transformed triglycerides. No significant correlations were observed (Supplementary Fig. S1). Furthermore, the hs-CRP levels between the groups were comparable (FET 0.3 mg/l [0.0, 0.5], Fresh-ET 0.4 mg/l [0.0, 0.5], NC 0.4 mg/l [0.1, 0.6], Kruskal–Wallis—*P* = 0.202), and no correlation was observed with VAT/SAT ratio (Supplementary Fig. S2).

### Intra-rater repeatability

The repeated analysis of twenty randomly selected MRI images of VAT, SAT, and NAT at the level of L_3_ showed, in general, good intra-rater repeatability with low mean differences (Supplementary Table S2).

## Discussion

In this study, we evaluated the abdominal distribution of adipose tissue including VAT, SAT and VAT/SAT ratio with the non-invasive reference standard of whole-abdominal MRI in 150 singletons at the age of 7–10 years born after FET, Fresh-ET and NC. With good intra-rater repeatability, the VAT/SAT ratio was significantly lower in children following FET as compared to children born after NC. The VAT/SAT ratio remained significantly lower in the FET group even after adjustment for confounding factors that have been well-described in the literature to be of potential importance. We found no statistically significant differences in VAT or SAT volumes, although we observed that children following Fresh-ET had numerically higher values of adipose compartments than those of the other two groups. Furthermore, the VAT/SAT ratio did not seem to be correlated with triglycerides or hs-CRP.

The abdominal adipose tissue has been widely accepted as an important modulator of the metabolic and cardiovascular system (Fox *et al.*, 2007; Ladeiras-Lopes *et al.*, 2017). In general, the VAT volume has been reported to be associated with cardiometabolic risk factors by the secretion of pro-inflammatory cytokines in both adults and children (Ouchi *et al.*, 2011; Tam *et al.*, 2011; Rainone *et al.*, 2016; Millar *et al.*, 2024). There is a consensus that excessive VAT may cause a state of chronic inflammation, thereby increasing the risk of cardiovascular disease (Koenen *et al.*, 2021; Rana and Neeland, 2022). In contrast, there are ambiguous data whether SAT has the same cardiometabolic properties as VAT and hence may be less pathogenic (McLaughlin *et al.*, 2011; Arner *et al.*, 2015). Thus, the VAT/SAT ratio may be a better surrogate marker to evaluate the cardiometabolic risk above and beyond BMI and VAT alone (Kaess *et al.*, 2012; Ladeiras-Lopes *et al.*, 2017).

It has long been hypothesized that children conceived after FET and Fresh-ET might be at increased risk of obesity in part mediated by the risk of being born large- and small-for-gestational-age, respectively (Hales and Barker, 2001; Hales and Ozanne, 2003; Maheshwari *et al.*, 2012; Kaul *et al.*, 2019; Derraik *et al.*, 2020; Luke *et al.*, 2021; Terho *et al.*, 2021a). In adulthood and early adolescence, obesity and high BMI are associated with increased risk of cardiovascular disease including hypertension and vascular stiffness (Khan *et al.*, 2018; Liu *et al.*, 2019; Liang *et al.*, 2021; Higgins *et al.*, 2022). Our previous data from the HiCART project did not support any indications of increased vascular stiffness and hypertension in ART children (Mizrak *et al.*, 2022b). In our latest work, however, we observed that children conceived after ART (both FET and Fresh-ET) had sympathetic predominance which may precede hypertension; yet no definite hypertension was observed. This observational sub-study on the HiCART cohort was conducted with fewer participants, including 8- to 12-year-old children (51% girls and 49% boys) conceived after FET (n = 34), Fresh-ET (n = 38), or NC (n = 33) (Mizrak *et al.*, 2024).

Reassuringly, the majority of existing studies found no significant difference in BMI when comparing FET, Fresh-ET, and NC (Wennerholm *et al.*, 1998; Ainsworth *et al.*, 2019; Terho *et al.*, 2021b; Asserhøj *et al.*, 2023). However, a recent population-based cohort study including 327 301 children aged 5–8 years, reported that children following FET (n = 1695, boys 47.1%) had a 1.5-fold increased risk of obesity as compared to Fresh-ET children (n = 11 791, boys 49.8%) (Laugesen *et al.*, 2023). Our study adds the new and newsworthy knowledge that children conceived by ART with FET or Fresh-ET do not have a different distribution of adipose tissue regarding VAT and SAT in comparison with NC children, and in fact, we even found a slightly lower VAT/SAT ratio in children conceived after FET suggesting, if anything, a possible lower cardiovascular risk. The latter finding, however, may have occurred due to statistical chance and needs further investigation in future studies. Furthermore, we observed that children conceived by Fresh-ET had numerically higher VAT and SAT values as compared to the other two groups, but without affecting the inflammatory state as evaluated by hs-CRP. Interestingly, even with comparable BMIs, we observed that children conceived by FET had significantly lower VAT/SAT ratio (as compared to NC children) indicating a possibly more favourable composition of abdominal adipose tissue without an increased pro-inflammatory state. However, the small albeit statistically significant difference observed in the VAT/SAT ratio among the three study groups probably has negligible clinical implications and, in fact, may be a chance finding. Thus, we do not believe that this small difference has any influence on the individual or population level.

Available data of biochemical tests on metabolic variables are ambiguous. Previous studies have reported elevated fasting plasma glucose and insulin resistance in children born after ART (Ceelen *et al.*, 2008; Cui *et al.*, 2020). However, data from the HiCART cohort did not confirm these findings concerning fasting plasma glucose levels (Mizrak *et al.*, 2022b; Asserhøj *et al.*, 2023). In 2022, Wijs *et al.* reported no significant differences in glucose, insulin, low-density lipoprotein, non-high-density lipoprotein, hs-CRP, or total cholesterol levels between ART and non-ART children. However, they found lower levels of triglycerides in young females following ART (Wijs *et al.*, 2022). In a systematic review, Guo *et al.* (2017) reported that ART children had significantly lower LDL cholesterol with a trend towards lower triglycerides and cholesterol and higher HDL. In the HiCART cohort, the glucose and lipid profiles were mostly comparable between the groups (Mizrak *et al.*, 2022b; Asserhøj *et al.*, 2024). In addition, the hs-CRP, as a marker of inflammation, was also comparable suggesting that ART children do not have a higher inflammatory state as compared to NC children. As an exploratory outcome, we investigated whether the VAT/SAT ratio was correlated with triglycerides and hs-CRP, and no significant observations were found. For adults, studies have reported significant correlations between both triglycerides and hs-CRP; however, these study populations were obese and had an atherogenic phenotype as compared to this study (Neeland *et al.*, 2013; von Krüchten *et al.*, 2022).

### Strengths and limitations

Previous studies on ART offspring have mostly evaluated the body composition by anthropometric measurements and/or DXA scans. In our study, we measured the metabolic active organ VAT and also SAT using the non-invasive reference standard of 3D whole-abdominal MRI which is an important strength of our study. In general, access to the healthcare system is equal for everyone in Denmark; thus, there are no differences in access to infertility treatment. A number of limitations were found in relation to this study. Intrinsic differences exist among the mothers in the three groups, such as the cause of infertility and socioeconomic status, which may contribute to variations in infant upbringing. Educational level can be considered as a surrogate measure for socioeconomic status, and our analysis revealed that mothers of children conceived by ART are slightly better educated than mothers of NC children. This makes the study subject to potential selection bias. The recruitment of children to the study depended on parental concern as parents of children born after ART may have a more genuine interest in the research findings and resourceful families may be more likely to participate in all three study groups. We examined this by conducting a non-participant analysis across all three groups and found FET participants to have higher birth weights, which may be associated with the same socioeconomic factors that influence participation. Moreover, maternal smoking during pregnancy was less prevalent among participants than among non-participants in the FET group, which may affect the results as maternal smoking is known to be related to low birth weight and altered cardiorespiratory responses (Knopik, 2009; Avşar *et al.*, 2021). In addition, we found that the participation rate was higher in FET and Fresh-ET compared to NC children, potentially leading to a risk of skewness between the groups. These results have been previously published (Mizrak *et al.*, 2022b). Only children living in the Capital and Zealand regions of Denmark were included which may make the population less generalizable. Moreover, the inflammatory state was assessed using hs-CRP; however, investigating additional inflammatory markers would hold greater relevance.

In the multivariable linear regression analysis, we accounted for birth weight and child BMI in a separate model. In the causal pathway, these variables are considered as mediators rather than confounders. We acknowledge that adjusting for other related child outcomes could lead to biased estimates. This was done to estimate the direct effect to the outcome (i.e. VAT/SAT ratio); however, the results did not change. Additionally, variables such as socioeconomic status, leisure time activities, dietary habits, and children’s participation in sports could potentially confound the results.

However, the absence of data pertaining to these variables precluded their inclusion in the multivariable regression analysis. Moreover, this is an exploratory observational cohort sub-study with no pre-specified primary outcome. Although various methods are available for handling multiple comparisons, we did not apply any multiple testing adjustments, given the exploratory nature of this study. We acknowledge that, without such corrections, some significant findings may occur by chance. Therefore, these findings should be interpreted cautiously until they are confirmed in an independent cohort. Lastly, this study is observational in nature, hence cannot conclude on the causality.

## Conclusion

With comparable inflammatory states, the VAT/SAT ratio may be slightly lower in FET children as compared to Fresh-ET and NC children; however, this is not believed to be of clinical relevance and may be a chance finding. Overall, our study results are reassuring for future ART treatments, as we did not find any indications of adverse abdominal adipose tissue distribution among children born following ART; if anything, rather the opposite.

## Supplementary Material

hoaf014_Supplementary_Data

## Data Availability

The data supporting this article will be made available upon reasonable request to the corresponding author.
