# Supplementary material for: Visceral and subcutaneous adipose tissue in children born after ART with frozen and fresh embryo transfers
Source: Hum Reprod Open. 2025 Mar 17;2025(2):hoaf014. doi: 10.1093/hropen/hoaf014 (PMC11975283; doi:10.1093/hropen/hoaf014)
Supplement: hoaf014_Supplementary_Data [file hoaf014_supplementary_data.zip › HRO-24-0128-R3-SuppTables1and2.docx]

**Supplementary Table S1. Quantification of abdominal adipose tissue at level L_3_**

| Variable | FET (n=50) | Fresh ET (n=50) | NC (n=50) | p-value | FET vs NC (crude)* | Fresh-ET vs NC (crude)* | FET vs Fresh-ET (crude)* |
| --- | --- | --- | --- | --- | --- | --- | --- |
| L3 SAT, median [IQR], cm^2^ | 35 [30, 47] | 38 [31, 60] | 33 [31, 43] | 0.532^a^ | -0.29 (-12.84; 12.26) | 3.71 (-8.85; 16.26) | -3.99 (-16.48; 8.49) |
| L3 superficial SAT, median [IQR], cm^2^ | 33 [30, 42] | 35 [30, 48] | 32 [30, 40] | 0.570^a^ | -0.87 (-6.94; 5.19) | 2.23 (-3.83; 8.30) | -3.11 (-9.14; 2.93) |
| L3 deep SAT, median [IQR], cm^2^ | 2 [0, 7] | 3 [0, 11] | 1 [0, 5] | 0.595^a^ | 0.59 (-6.16; 7.33) | 1.47 (-5.27; 8.22) | -0.89 (-7.60; 5.83) |
| L3 VAT, median [IQR], cm^2^ | 12 [8, 20] | 16 [11, 25] | 14 [10, 21] | 0.095^a^ | -1.98 (-6.93; 2.96) | 0.63 (-4.31 5.58) | -2.62 (-7.54; 2.30) |
| L3 VAT/SAT ratio, mean (SD) | 0.33 (0.13) | 0.39 (0.14) | 0.39 (0.12) | **0.035**^b^ | **-0.06 (-0.11; -0.01)** | -0.00 (-0.06; 0.05) | **-0.06 (-0.107; -0.004)** |
| L3 TAT, median [IQR], cm^2^ | 49 [37, 62] | 54 [43, 85] | 48 [41, 62] | 0.373^a^ | -2.27 (-19.10; 14.55) | 4.34 (-12.49; 21.17) | -6.61 (-23.36; 10.13) |
| L3 NAT, median [IQR], cm^2^ | 201 [183, 223] | 202 [182, 219] | 196 [183, 212] | 0.437^a^ | 9.21 (-1.62; 20.04) | 6.24 (-4.59; 17.06) | 2.97 (-7.80; 13.75) |

Values are showed as mean (SD, standard deviation) for normally distributed or median [IQR, interquartile ranges] for non-normally distributed data. ^a^ Data were analysed using Kruskal-Wallis. ^b^ Data were analysed using ANOVA. Boldface indicates significant values. ^*^ Pairwise comparison of groups by calculating mean differences with 95% confidence interval for continuous variables (using univariable linear regression model).

VAT, visceral adipose tissue; SAT, subcutaneous adipose tissue; TAT, total adipose tissue; NAT, non-adipose tissue; FET, frozen embryo transfer; Fresh-ET, fresh embryo transfer; NC, natural conception; L3, lumbar spine level L3.

**Supplementary Table S2. Intra-rater repeatability** **at the level of L_3_**

| Bland Altman | Mean difference | 95% limits of agreement |
| --- | --- | --- |
| L3 SAT, cm2 | -0.1 | (-0.98; 0.79) |
| L3 VAT, cm2 | -0.1 | (-2.34; 2.13) |
| L3 NAT, cm2 | 0.05 | (-2.92; 2.86) |

Presented data are mean difference and 95% limits of agreement of Bland-Altman analysis. VAT, visceral adipose tissue;

SAT, subcutaneous adipose tissue; NAT, non-adipose tissue; L3, lumbar spine level L3.
