# Supplementary material for: Visceral and subcutaneous adipose tissue in children born after ART with frozen and fresh embryo transfers
Source: Hum Reprod Open. 2025 Mar 17;2025(2):hoaf014. doi: 10.1093/hropen/hoaf014 (PMC11975283; doi:10.1093/hropen/hoaf014)
Supplement: hoaf014_Supplementary_Data [file hoaf014_supplementary_data.zip › Supplementary Figure S2 - Word.docx]

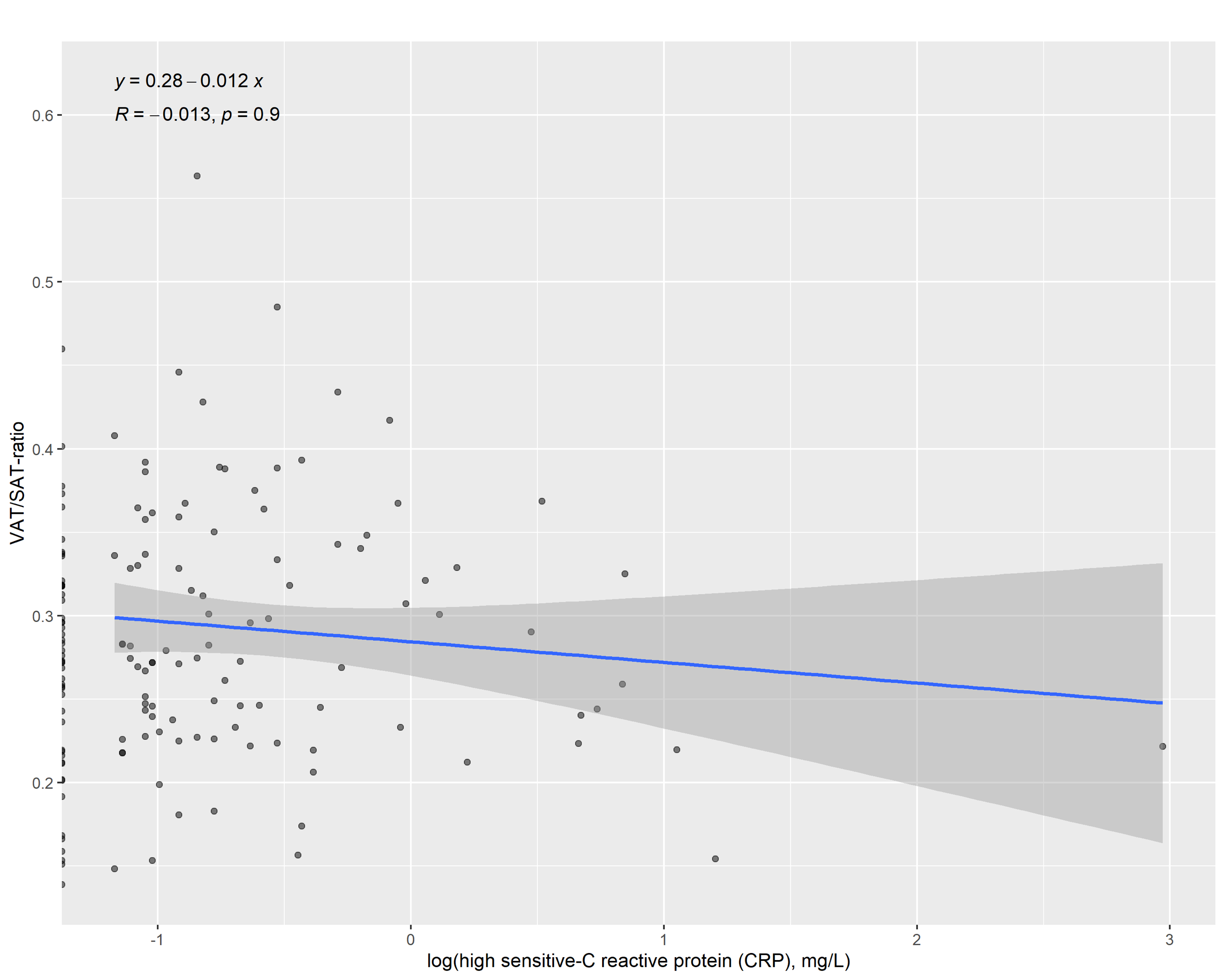


**Supplementary Figure S2. Correlation between VAT/SAT-ratio and Log(hs-CRP).** The correlation coefficient R is estimated using the Spearman method. VAT, visceral adipose tissue; SAT, subcutaneous adipose tissue; Log, logarithmic; hs-CRP, high-sensitivity C-reactive protein.
